# Spiralosides A–C, Three New C_27_-Steroidal Glycoalkaloids from the Fruits of *Solanum spirale*

**DOI:** 10.1007/s13659-016-0103-9

**Published:** 2016-06-18

**Authors:** Dan Li, Yun-Li Zhao, Xu-Jie Qin, Lu Liu, Xing-Wei Yang, Ying-Ying Chen, Bei Wang, Xin Wei, Ya-Ping Liu, Xiao-Dong Luo

**Affiliations:** State Key Laboratory of Phytochemistry and Plant Resources in West China, Kunming Institute of Botany, Chinese Academy of Sciences, Kunming, 650201 China; University of Chinese Academy of Sciences, Beijing, 100039 China

**Keywords:** Solanaceae, *Solanum spirale*, Spiralosides A–C

## Abstract

**Abstract:**

Three new C_27_-steroidal glycoalkaloids, spiralosides A–C (**1**–**3**), were obtained from the total alkaloids of *Solanum spirale* by chromatographic methods. On the basis of spectroscopic evidence, spiralosides A–C were elucidated as (22*R*,25*S*)-22,26-epiminocholest-5-ene-3*β*,16*α*-diol-*N*-acetyl-3-*O*-*α*-l-rhamnopyranosyl-(1→4)-*β*-d-glucopyranosyl (**1**), (22*R*,25*S*)-22,26-epiminocholest-5-ene-3*β*,16*α*-diol-*N*-acetyl-3-*O*-*β*-d-glucopyranosyl (**2**), (22*R*,25*S*)-22,26-epiminocholest-3*β*,16*α*-diol-*N*-acetyl-3-*O*-*β*-d-glucopyranosyl (**3**), respectively. The total alkaloids of *S. spirale* have been screened for their antitussive and expectorant effects in intact animal model.

**Graphical Abstract:**

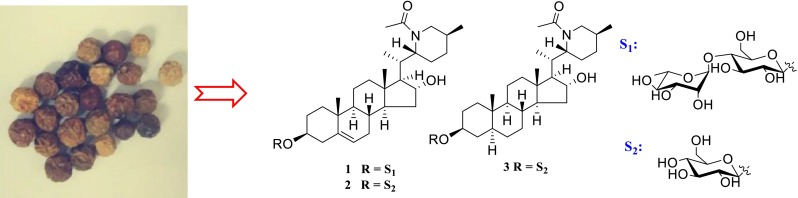

**Electronic supplementary material:**

The online version of this article (doi:10.1007/s13659-016-0103-9) contains supplementary material, which is available to authorized users.

## Introduction

*Solanum spirale* (Solanaceae), an erect shrub, is widely distributed in Yunnan, Guangxi and Hunan provinces of China. It usually grows forest-shrub edge and wasteland at the altitude between 500 and 1900 m [1]. *S. spirale*, popularly named “Li Fei San”, has been used in “Yi” ethnopharmacy as anti-tussive and anti-inflammatory agent historically. Besides, its tender leaves and fruits can be edible as a wild vegetable by “Dai” ethnopharmacy [2]. Previously, the reported phytochemical studies of *S. spirale* led to the isolation of a series of components, such as oil components, steroidal saponins, steroidal alkaloids and steroidal glycoalkaloids with antioxidant activity, and significant antibacterial activity and cytotoxicity [3–8]. The steroidal alkaloid glycosides from *Solanum* genus possess extensive activities including anticancer [9, 10], anti-acetylcholinesterase [11, 12], anticholesterol [13, 14], and antifungal [15] properties. In the course of searching for bioactive steroidal alkaloid glycosides from the fruits of *S. spirale*, three new steroidal glcoalkaloids named spiralosides A–C (**1**–**3**) were isolated. The total alkaloids were evaluated anti-tussive activity against ammonia liquor induced damage and the expectorant effects used by phenol red secretion test in mice. Reported herein are the isolation, structural elucidation, and the anti-tussive activities of the total alkaloids.

## Result and Discussion

**Fig. 1:**
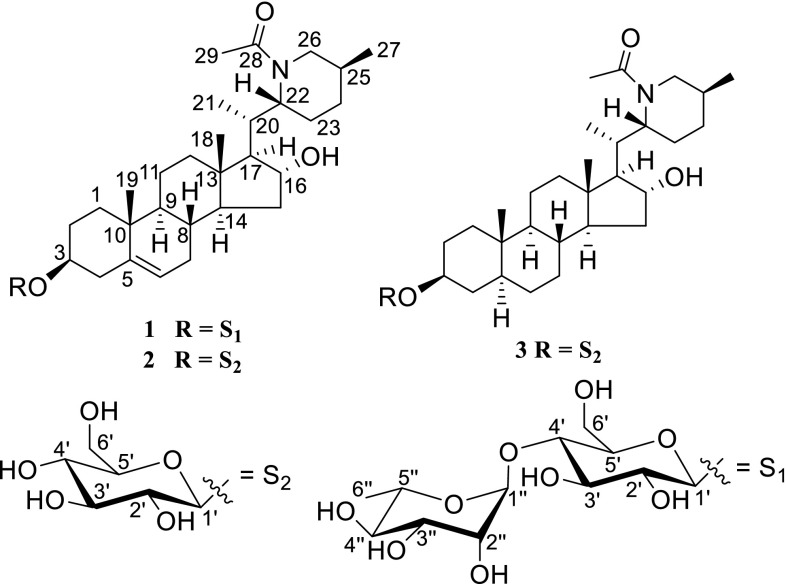
The chemical structures of spiralosides A–C (**1**–**3**)

**Fig. 2:**
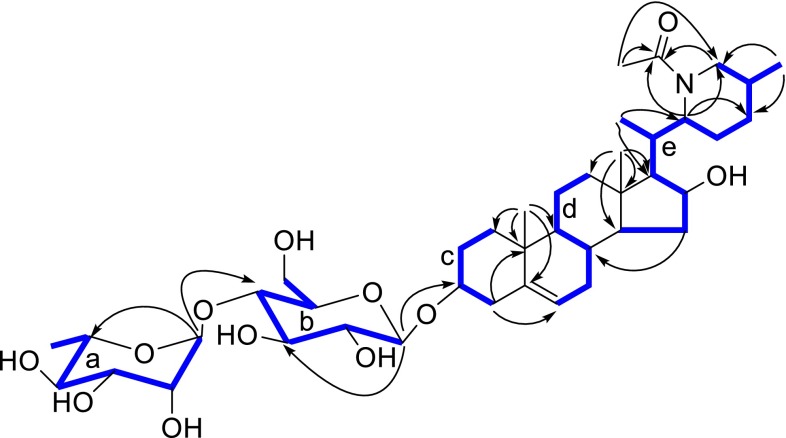
Key ^1^H-^1^H COSY () and HMBC () correlations of **1**

Compound **1** was obtained as a white, amorphous powder; $[\alpha ]_{\text{D}}^{23}$ −46.6 (*c* 0.03, MeOH). It displayed a positive reaction to Dragendorff’s reagent and gave the molecular formula of C_41_H_67_NO_12_ by HRESIMS at *m/z* 788.4553 [M+Na]^+^ (calcd for 788.4561), corresponding to nine degrees of unsaturation. The ^1^H-, ^13^C-NMR and DEPT spectra displayed two sugar units on basis of three proton signals at *δ*_H_ 4.36 (1H, d, *J* = 7.8 Hz), 4.81 (1H, br s), and 1.23 (3H, d, *J* = 6.2 Hz), two anomeric carbons [*δ*_C_ 102.3 (d), and 102.9 (d)], a methyl group (*δ*_C_ 17.8), a methylene carbon (*δ*_C_ 62.1), and other 8 methines signals between *δ*_C_ 70.6 and δ_C_ 79.9. The coupling constant (*J* = 7.8 Hz) of the anomeric proton at *δ*_H_ 4.36 (1H, d, H-1) indicated the *β*-configuration of the glucosyl residues [16]. Likewise the other anomeric configuration of the rhamnopyranosyl was confirmed as *α*-orientated on the basis of the chemical shift values of C-3″ (*δ*_C_ 72.2), C-5″ (*δ*_C_ 70.6) with those of the corresponding carbons of methyl α- and *β*-rhamnopyranoside [17].The identification of the sugar residues were continued by hydrolysis with 10 % HCl to afford d-glucose and l-rhamnose, which were confirmed by GC chromatographic analysis of their l-cysteine methyl ester-TMS derivates. Besides of two sugar units, ^13^C-NMR and DEPT spectra also showed 29 carbons, five methyl groups, ten methylenes, ten methines, and four quaternary carbons (Table 1). Comparison of above data with those of capsimine [18] and baikeine [19], pingbeinine [20] showed that the aglycone of **1** was similar to capsimine with exception of an additional acetyl group at *δ*_C_ 22.2 (q) and 172.4 (s) in **1** (Fig. 1). This acetyl group (*δ*_C_ 22.2, q and 172.4, s) was located at N in last ring by correlations of *δ*_H_ 2.08 (3H, s, H-29) with *δ*_C_ 172.4 (s, C-28) and 50.7 (t, C-26), of *δ*_H_ 3.60 (1H, overlap, H-26a), 2.91 (1H, t, *J* = 12.4 Hz, H-26b), and 4.46 (1H, dt, *J* = 6.1, 6.0 Hz, H-22) with δ_C_ 172.4 (s, C-28). Signal at *δ*_H_ 4.22 (1H, t, *J* = 7.4 Hz) corresponding to *δ*_C_ 76.3 (t) in its HSQC spectrum showed cross peaks with *δ*_C_ 61.9 (d, C-17), 55.2 (d, C-14), and 37.4 (t, C-15), which suggested that the hydroxyl substitute at C-16. The other oxygenic proton signal at *δ*_H_ 3.53 (1H, m) placed at C-3, which was confirmed by HMBC correlations of *δ*_H_ 1.84 (1H, m, H-1a), 1.03 (1H, d, *J* = 4.3 Hz, H-1b), 2.38 (1H, dd, *J* = 2.1, 13.1 Hz, H-4a), and 2.22 (1H, t, *J* = 12.4 Hz, H-4b) with *δ*_C_ 79.9 (d, C-3) (Fig. 2). The glycositatic position was unambiguously ascribed to be at C-3 from the HMBC correlation of *δ*_H_ 4.36 (1H, d, *J* = 7.8 Hz, H-1′) with C-3 (*δ*_C_ 79.9). The ^1^H-^1^H COSY spectrum of **1** displayed five partial fragments **a**–**e** (Fig. 2). The signals of fragments **a** and **b** were from δ_H_ 4.36 (1H, d, *J* = 7.8 Hz, H-1′) to δ_H_ 4.76 and 3.62 (1H each, H-6′), from δ_H_ 4.81 (1H, br s, H-1″) to δ_H_ 1.23 (3H, q, *J* = 7.8 Hz, H-6″), respectively, were further suggested a rhamnose and a glucose appeared. On the basis of δ_H_ 4.81 (1H, br s, H-1″) correlating with C-4′ (δ_C_ 79.6), the presence of a rhamnopyranosyl (1→4)-glucopyranosyl moiety was deduced (Fig. 2).

**Table 1:** ^1^H and ^13^C NMR Spectroscopic Data of Compounds **1**–**3** (methanol-*d*_4_ *δ* in ppm)

Position	**1**		**2**		**3**	
	*δ*_H_	*δ*_C_	*δ*_H_	*δ*_C_	*δ*_H_	*δ*_C_
1a	1.84 (1H, m)	38.5 (t)	1.83 (1H, m)	38.5 (t)	1.59 (1H)^*o1*^	37.4 (t)
1b	1.03 (1H, d, 4.3)		1.04 (1H, br s)		1.47(1H)^*o2*^	
2a	1.89 (1H)^*o1*^	30.7 (t)	1.89 (1H)^*o1*^	30.7 (t)	1.83 (1H)^*o3*^	30.5 (t)
2b	1.57 (1H)^*o2*^		1.57 (1H)^*o2*^		1.73 (1H)^*o4*^	
3	3.53 (1H, m)	79.9 (d)	3.54 (1H, m)		3.66 (1H, m)	79.3 (d)
4a	2.38 (1H, dd, 2.1, 13.1)	39.7 (t)	2.38 (1H, dd, 2.4, 13.2)		1.66 (1H)^*o5*^	38.2 (t)
4b	2.22 (1H, t, 12.4)		2.21 (1H, t, 12.6)		0.95 (1H, m)	
5		142.0 (s)		142.0 (s)	1.05 (1H, m)	46.0 (d)
6a 6b	5.34 (1H, br s)	122.6 (d)	5.34 (1H, br s)	122.7 (d)	1.49 (1H)^*o2*^ 1.27 (1H)^*o6*^	30.2 (t)
7a	1.92 (1H)^*o1*^	33.0 (t)	1.93 (1H)^*o1*^	33.0 (t)	1.62 (1H)^*o5*^	33.3 (t)
7b	1.53 (1H)^*o2*^		1.53 (1H)^*o2*^		0.91 (1H,m)	
8	1.44 (1H)^*o3*^	32.5 (d)	1.45 (1H)^*o3*^	32.5 (d)	1.33 (1H, m)	36.0 (d)
9	0.93 (1H, d, 4.5)	51.7 (d)	0.93 (1H, d, 5.4)	51.6 (d)	0.65 (1H, m)	55.8 (d)
10		37.9 (s)		37.9 (s)		36.7 (s)
11a	1.48 (1H)^*o3*^	21.7 (t)	1.48 (1H)^*o3*^	21.7 (t)	1.58 (1H)^*o1*^	21.9 (t)
11b	1.30 (1H)^*o4*^		1.23(1H)^*o4*^		1.46 (1H)^*o2*^	
12a	1.92 (1H)^*o1*^	41.2 (t)	1.91 (1H)^*o1*^	41.1 (t)	1.87 (1H)^*o3*^	41.5 (t)
12b	1.23 (1H)^*o4*^		1.23 (1H)^*o4*^		1.18 (1H, m)	
13		44.9 (s)		44.8 (s)		45.2 (s)
14	1.31 (1H)^*o4*^	55.3 (d)	1.31 (1H)^*o4*^	55.3 (d)	1.30 (1H, m)	55.0 (d)
15a	1.61 (1H)^*o5*^	37.5 (t)	1.61 (1H)^*o5*^	37.5 (t)	1.64 (1H)^*o5*^	35.3 (t)
15b	1.49 (1H)^*o3*^		1.47 (1H)^*o3*^		1.55 (1H)^*o3*^	
16	4.22 (1H, dt, 7.0, 7.4)	76.3 (d)	4.22 (1H, t, 7.4)	76.3 (d)	4.22 (1H, t, 7.4)	76.3 (d)
17	1.18 (1H, dd, 7.0, 12.4)	61.9 (d)	1.18 (1H, t, 7.8)	62.2 (d)	1.16 (1H, t, 7.2)	62.2 (d)
18	0.72 (3H, s)	14.7 (q)	0.72 (3H, s)	14.7 (q)	0.69 (3H, s)	14.8 (q)
19	0.98 (3H, s)	19.8 (q)	0.98 (3H, s)	19.8 (q)	0.79 (3H, s)	13.1 (q)
20	2.27 (1H, ddq, 6.0, 7.1, 12.4)	35.7 (d)	2.27 (1H, ddq, 6.0, 7.1, 14.2)	35.8 (d)	2.27 (1H, ddq, 6.1, 7.2, 14.4)	35.8 (d)
21	0.96 (3H, d, 7.1)	15.1 (q)	0.97 (3H, d, 7.1)	15.1 (q)	0.94 (3H, d, 6.6)	15.1 (q)
22	4.46 (3H, ddd, 6.0, 6.0, 12.0)	55.4 (d)	4.46 (3H, ddd, 6.0, 6.0, 12.1)	55.4 (d)	4.44 (3H, ddd, 6.1, 6.1, 12.6)	55.5 (d)
23a	1.72 (1H)^*o6*^	22.2 (t)	1.72 (1H)^*o6*^	22.3 (t)	1.72 (1H)^*o4*^	22.1 (t)
23b	1.62 (1H)^*o5*^		1.61 (1H)^*o5*^		1.61 (1H)^*o5*^	
24a	1.53 (1H)^*o2*^	28.6 (t)	1.52 (1H)^*o2*^	28.6 (t)	1.50 (1H)^*o2*^	28.6 (t)
24b	1.29 (1H)^*o4*^		1.29 (1H)^*o4*^		1.28 (1H)^*o6*^	
25	1.75 (1H)^*o6*^	31.3 (d)	1.75 (1H)^*o6*^	31.3 (d)	1.73(1H)^*o4*^	31.2 (d)
26a	3.60 (1H)^*o7*^	50.7 (t)	3.59 (1H)^*o7*^	50.7 (t)	3.58 (1H)^*o7*^	50.8 (t)
26b	2.91 (1H, t, 12.4)		2.91 (1H, t, 13.8)		2.90 (1H, t, 13.8)	
27	0.89 (3H, d, 6.6)	19.4 (q)	0.89 (3H, d, 6.6)	19.5 (q)	0.88 (3H, d, 6.6)	19.5 (q)
28		172.4 (s)		172.5 (s)		172.4 (s)
29	2.08 (3H, s)	22.2 (q)	2.07 (3H, s)	22.3 (q)		
1′	4.36 (1H, d, 7.8)	102.3 (d)	4.34 (1H, d, 7.8)	102.4 (d)	4.34 (1H, d, 7.8)	102.2 (d)
2′	3.15 (1H, t, 8.5)	75.2 (d)	3.10 (1H, t, 8.4)	75.1 (d)	3.09 (1H, t, 7.8)	75.1 (d)
3′	3.42 (1H, t, 8.9)	76.7 (d)	3.31 (1H, t, 8.4)	78.0 (d)	3.30 (1H, t, 8.4)	78.1 (d)
4′	3.49 (1H, t, 9.1)	79.6 (d)	3.23 (1H)^*o8*^	77.8 (d)	3.21 (1H)^*o8*^	77.8 (d)
5′	3.29 (1H)^*o8*^	76.8 (d)	3.22 (1H)^*o8*^	71.5 (d)	3.22 (1H)^*o8*^	71.6 (d)
6′a	3.76 (1H, d, 12.0)	62.1 (t)	3.81 (1H, d, 12.0)	62.7 (t)	3.81 (1H, d, 12.0)	62.8 (t)
6′b	3.62 (1H)^*o7*^		3.61 (1H)^*o7*^		3.60 (1H)^*o7*^	
1″	4.81 (1H, br s)	102.9 (d)				
2″	3.80 (1H, br s)	72.4 (d)				
3″	3.59 (1H)^*o7*^	72.2 (d)				
4″	3.36 (1H, t, 9.5)	73.7 (d)				
5″	3.93 (1H, dq, 6.2, 12.4)	70.6 (d)				
6″	1.23 (3H, d, 6.2)	17.8 (q)				

^1^H-NMR Recorded at 600 MHz, ^13^C-NMR Recorded at 150 MHz

^*o1*–*o7*^Overlapped signals

^*o8*^Overlapped CD_3_OD

**Fig. 3:**
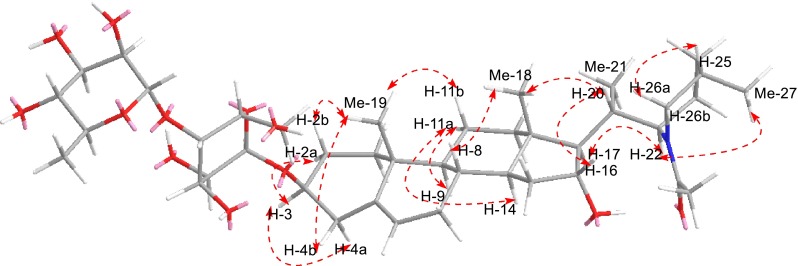
Key ROESY () correlations of **1**

The ROESY correlations of Me-19/H-4b/H-2b/H-11b/H-8, H-3/H-2a, and H-11a/H-9/H-14 indicated the *α*-orientation of H-3, H-9 and H-14, *β*-orientation of H-8 (Fig. 3). ROESY corrections of Me-18 with H-20, of H-20 with H-16 positioned *α*-orientation for both Me-21 and OH-16, *β*-orientation for H-20. ROESY corrections of Me-18 with H-20 also indicated a 17*β*-side chain, as well as on the biogenetical derivation of C-27 steroidal alkaloids [21]. And a missing effect between H-17 and H-20 displayed, at least approximately, antiperiplanar positions of H-17 and H-20. The coupling constant ^3^*J*_17,20_ = 12.4 Hz was in agree with this assumption [7]. Furthermore, the coupling constant ^3^*J*_20,22_ = 6.0 Hz suggested that they should be on the same side and the C-22 of configuration is *R* [22]. Besides, ROESY corrections of H-22 with H-23b, of Me-27 with H-23a indicated H-22 and H-25 both are axial bonds and the C-25 of configuration is *S*. So compound **1** was determined as (22*R*,25*S*)-22,26-epiminocholest-5-ene-3*β*,16*α*-diol-*N*-acetyl-3-*O*-*α*-l-rhamnopyranosyl-(1→4)-*β*-d-glucopyranosyl, named spiraloside A. All of the signals of ^1^H- and ^13^C-NMR were assigned by HSQC, HMBC, ^1^H-^1^H COSY and ROESY spectra (Table 1).

Compound **2** was isolated as a white, amorphous powder; $[\alpha ]_{\text{D}}^{23}$ −43.9 (*c* 0.03, MeOH). The molecular formula C_35_H_57_NO_8_ was established by the HRESIMS quasimolecular ion at *m/z* 642.3975 [M+Na]^+^ (calcd for *m/z* 642.3976), indicating eight degree of unsaturation. Its IR spectrum revealed the presence of hydroxyls (3440 cm^−1^) and an acylamino group (1630 cm^−1^). The 1D-NMR data (Table 1) were similar to those of **1** except the absence of the rhamnopyranosyl moiety, as supported by the HMBC correlations of *δ*_H_ 4.34 (1H, d, J = 7.8 Hz, H-1′) with *δ*_C_ 79.8 (d, C-3). Other parts of the structure were identical to those of **1** by detailed analysis of 2D-NMR and acid hydrolysis of **2**. Consequently, spiraloside B (**2**) was determined as (22*R*,25*S*)-22,26-epiminocholest-5-ene-3*β*,16*α*-diol-*N*-acetyl-3-*O*-*β*-d-glucopyranosyl (Fig. 1).

Spiraloside C (**3**), obtained as an amorphous powder, had the molecular formula of C_35_H_59_NO_8_ as revealed by HRESIMS (calcd for C_35_H_59_NO_8_ at *m/z* 621.4241), corresponding to seven degrees of unsaturation. The IR data showed the presence of hydroxys (3440 cm^−1^) and an acylamino group (1619 cm^−1^). Comparison of 1D-NMR data of **3** with those of **2** (Table 1), showed that the two compounds were similar with exception of the absence of a double bond at C-5/6 in **3**, which was suggested by the HMBC correlations of *δ*_H_ 0.79 (3H, s, H-19) with *δ*_C_ 46.0 (d, C-5), and 1.27 (1H, m, H-6b) with *δ*_C_ 46.0 (d, C-5), as well as one less degree of unsaturation. The ROESY correlations of Me-19/H-2b, H-3/H-2a/H-5 indicated the α-orientation of H-3 and H-5. Other parts of the structure were identical to those of **2**, by detailed analyses of 2D-NMR and acid hydrolysis of **3**. Thus, spiraloside C (**3**) was determined as (22*R*,25*S*)-22,26-epiminocholest-3*β*,16*α*-diol-*N*-acetyl-3-*O*-*β*-d-glucopyranosyl (Fig. 1).

The total alkaloids of *S. spirale* have been screened for the protective anti-tussive effect against ammonia liquor induced cough and the protective expectorant activity used by phenol red secretion test in mice. The results showed that the total alkaloids exhibited an inhibited tendency on antitussive in mice (Tables 2, 3).

**Table 2:** Effect of the total alkaloids on the ammonia liquor induced cough in mice

Groups	Dose (mg/kg)	Treatment	Frequency of cough	Inhibition (%)
Control	–	ig	23.5 ± 10.3	–
Codeine phosphate	30	ig	9.2 ± 4.1**	60.9
The total alkaloids	10	ig	16.4 ± 4.8	30.2

Values expressed as mean ± SEM (n = 10)

** *P* < 0.01 for comparison of treated groups with control

**Table 3:** Effects of the total alkaloids on tracheal phenol red output in mice

Groups	Dose (mg/kg)	Treatment	Absorbance	Phenol red secretion (μg/mL)
Control	–	ig	0.140 ± 0.058	2.66 ± 1.64
Carbocisteine	10	ig	0.214 ± 0.063*	4.75 ± 1.77*
The total alkaloids	10	ig	0.175 ± 0.053	3.67 ± 1.49

Values expressed as mean ± SEM (n = 10)

* *P* < 0.05 for comparison of treated groups with control

Compounds **1**–**3** were evaluated for their cytotoxicity against five human cancer cell lines using the MTT method as reported previously [23]. Cisplation (sigma, USA) was used as the positive control. Unfortunately, the results showed that all compounds were inactive (IC_50_ values >40 µM).

## Experimental Section

### Plant Material

Air-dried fruits of *S. spirale* were collected in December 2014 from Shuangbai county, Yunnan province, P.R china, and identified by one of the author Yun-Li Zhao, Kunming Institute of Botany, Chinese Academy of Sciences. A voucher specimen (No. 20141225) was deposited at the State Key Laboratory of Phytochemistry and Plant Resources in West China, Kunming Institute of Botany, Chinese Academy of Sciences.

### Extraction and Isolation

The air-dried fruits from *S. spirale* (5 kg) were crushed and extracted with 20 L 90 % MeOH for five times under reflux for a total 3 h, and then combined extract was concentrated under reduced pressure to afford an extract. The extract was partitioned between EtOAc and 0.5 % HCl solution, getting a nor-alkaloid fraction. The acidic water-soluble, adjusted pH to 9–10, was extracted with EtOAc to give total alkaloids and water-soluble fraction. The total alkaloid fraction (84 g) was subjected to silica gel column (200–300 mesh; gradient CHCl_3_–MeOH 20:1, 10:1, 8:1, 6:1, 4:1, 3:1, 1:1, 0:1 v/v) to afford fractions 1–6, respectively. Fr.3 (5 g) was chromatographed over silica gel CC (200–300 mesh), eluted with repeated CHCl_3_:MeOH:H_2_O (10:1:0.1 to 0:1:1) to obtain three subfractions (Fr.3-1 to Fr.3-3). Fr.3-1 (66 mg) was then separated by repeated RP-18 CC with MeOH:H_2_O (5:5 to 10:0) and Sephdex LH-20 (MeOH) to give **3** (9 mg). Fr.4 (8.4 g) was submitted to RP-18 CC and elicited with MeOH:H_2_O (1:9 to 8:2) to afford five subfractions (Fr.4-1 to Fr.4-5). Fr.4-3 (900 mg) was chromatographed over silica gel CC (200–300 mesh), eluted with CHCl_3_:MeOH:H_2_O (8:2:0.2 to 0:1:1) to obtain three subfractions (Fr.4-3-1 to Fr.4-3-3). Fr.4-3-2 (35 mg) was further purified by Sephdex LH-20 (MeOH/H_2_O, 1:9) to obtain **2** (10 mg). Fr.6 (6 g) was separated by RP-18 CC and elicited with MeOH:H_2_O (1:9 to 10:0) to yield five subfractions (Fr.6-1 to Fr.6-5). Fr.6-3 (1.5 g) was further separated by repeated silica gel with CHCl_3_:MeOH:H_2_O (7:3:0.3 to 0:1:1), then followed by Sephdex LH-20 with MeOH:H_2_O (1:9 to 0:1) to obtained three subfractions (Fr.6-3-1 to Fr.6-3-3), Fr.6-3-2 (25 mg) was further purified by semi-preparative HPLC (28 % CH_3_CN) to yield **1** (13 mg).

Spiraloside A (**1**): White amorphous powder; $[\alpha ]_{\text{D}}^{23}$ −46.6 (*c* 0.03, MeOH); IR (KBr) *v*_max_ 3441, 2934, 2870, 1623, 1442, 1383, 1343, 1266, 1127, 1056, 1038, 809 cm^−1^; ^1^H, ^13^C-NMR spectroscopic data see Table 1; Positive ESIMS m/z 788 [M+Na]^+^; HREIMS *m/z* 788.4553 [M+Na]^+^ (calcd for C_41_H_67_NO_12_, 788.4561).

Spiraloside B (**2**): white amorphous powder; $[\alpha ]_{\text{D}}^{23}$ −43.9 (*c* 0.03, MeOH); IR (KBr) *v*_max_: 3440, 2969, 2928, 1630, 1553, 1445, 1383, 1267, 1197, 1079, 1046 cm^−1^; ^1^H, ^13^C-NMR spectroscopic data see Table 1; Positive ESIMS *m/z* 642 [M+Na]^+^; HREIMS *m*/*z* 642.3975 [M+Na]^+^ (calcd for C_35_H_57_NO_8_, 642.3976).

Spiraloside C (**3**): white amorphous powder;$[{{\alpha }}]_{\text{D}}^{24}$ −30.1 (*c* 0.09, MeOH); IR (KBr) *v*_max_: 3427, 2928, 2858, 1727, 1619, 1444, 1382, 1075, 1046 cm^−1^; ^1^H, ^13^C-NMR spectroscopic data see Table 1; Positive ESIMS *m/z* 644 [M+Na]^+^; HREIMS *m*/*z* 621.4231 [M+H]^+^ (calcd for C_35_H_59_NO_8_, 621.4241).

### Acid Hydrolysis of compounds **1**–**3** and GC Analysis

Compounds **1**–**3** (each 3 mg) were refluxed with 2 M HCl (1,4 dioxane/H_2_O 1:1, 2 mL) on water bath for 2 h. After cooling, the reaction mixture was neutralized with 1 M NaOH. The reaction mixture was extracted with CHCl_3_ (3 × 5 mL). The aqueous layer was evaporated to dryness. The dried residue was dissolved in 1 mL anhydrous pyridine and treated with l-cysteine methyl ester hydrochloride (1.5 mg) stirred at 60 °C for 1 h. Trimethylsilylimidazole (1.0 mL) was added to the reaction mixtures, and they were kept at 60 °C for 30 min. The supernatants (4 μL) were analyzed by GC, respectively, under the following conditions: H_2_ flame ionization detector. Column: 30QC2/AC-5 quartz capillary column (30 m × 0.32 mm). Column temperature: 180–280 °C with the rate of 3 °C/min, and the carrier gas was N_2_ (1 mL/min) injector temperature: 250 °C; and split ratio: 1/50. Peaks of the hydrolysate were detected by comparison with retention times of authentic samples of d-glucose and L-rhamnose after treatment with trimethyl–chlorosilane (TMCS) in pyridine. The absolute configurations of the compounds **1**–**3** were determined by comparison of the retention times of the corresponding derivatives with those of standard d-glucose and L-rhamnose giving a single peak at 19.01 and 15.43 min, respectively.

### Animals

ICR mice of either sex (20–22 g) were purchased from Kunming Medical College (License number SYXK2014-0004). All animals were housed at room temperature (20–25 ^°^C) and constant humidity (40–70 %) under a 12 h light–dark cycle in SPF grade laboratory. The animal study was performed according to the international rules considering animal experiments and the internationally accepted ethical principles for laboratory animal use and care.

### Anti-tussive Activity Assay

ICR mice of either sex weighing 21–24 g were divided randomly, 10 mice per group. The negative control of animals was treated with distilled water orally, and the positive control was treated with codeine phosphate, the remaining groups treated were with test samples respectively. Anti-tussive activity was investigated on a classical mouse cough model induced by ammonia liquor [24]. Briefly, each mouse was placed in a 300 mL special glass chamber and exposed to 40*μ*L 25 % NH_4_OH. The cough frequency produced during 2 min exposure period was counted. In the second assay for alkaloids, cough frequency and latent period of cough were recorded.

### Expectorant Effect Assessment

The procedures were performed as described previously [25]. Male and female mice were randomly allotted and treated with a single dose 30 min before intraperitoneal injection of phenol red solution (5 % in saline solution, w/v, 0.1 mL/10 g body weight). Mice were sacrificed by cervical dislocation 30 min after application of phenol red. After dissected free from adjacent organs, the trachea was removed from the thyroid cartilage to the main stem bronchi and put into 2 mL normal saline immediately. After ultrasonic for 5 min, 0.1 mL of 1 M NaOH solution was added to the saline and optical density of the mixture were measured at 546 nm using enzyme standard instrument.

### Cytotoxicity Assay

Five human cancer cell lines, lung cancer A-549, human myeloid leukemia HL-60, hepatocellular carcinoma SMMC-7721, breast cancer MCF-7, and colon cancer SW480 cells, were used in the cytotoxic assay. All the cells were cultured in RPMI-1640 or DMEM medium (Hyclone, USA), supplemented with 10 % fetal bovine serum (Hyclone, USA) in 5 % CO_2_ at 37 °C. The cytotoxicity assay was performed according to the MTT (3-(4,5-dimethylthiazol-2-yl)-2,5-diphenyltetrazolium bromide) method in 96-well microplates [26]. Briefly, 100 μL of adherent cells was seeded to each well of a 96-well cell culture plates and allowed to adhere for 12 h before drug addition, while suspended cells were seeded just before drug addition with an initial density of 1 × 10^5^ cells/mL in 100 *μ*L of medium. Each tumor cell line was exposed to the test compound dissolved in DMSO at concentrations of 0.064, 0.32, 1.6, 8, and 40 μM in triplicates for 48 h, with cisplatin (Sigma, USA) as a positive control. After compound treatment, cell viability was detected, and the cell growth curve was graphed. The IC_50_ value was calculated by Reed and Muench’s method [27].

## Electronic supplementary material

Below is the link to the electronic supplementary material.

Supporting Information Available: bioassay, 1D, 2D-NMR, MS and IR spectra of spiralosides A–C (**1**–**3**), these supplementary materials are available in the online version of this article and is accessible for authorized users (PDF 1341 kb)
