# Supplementary material for: Spiralosides A–C, Three New C27-Steroidal Glycoalkaloids from the Fruits of Solanum spirale
Source: Nat Prod Bioprospect. 2016 Jun 18;6(4):225–31. doi: 10.1007/s13659-016-0103-9 (PMC4940254; doi:10.1007/s13659-016-0103-9)

Supplementary data

Spiralosides A–C, Three New C_{27}-Steroidal Glycoalkaloids from the Fruits of *Solanum spirale*

Dan Li [a,b], Yun-Li Zhao [a], Xu-Jie Qin [a], Lu Liu [a,b], Xing-Wei Yang [a], Ying-Ying Chen [a,b], Bei Wang [a,b], Xin Wei [a,b], Ya-Ping Liu [a,*], Xiao-Dong Luo [a,*]

a. State Key Laboratory of Phytochemistry and Plant Resources in West China, Kunming Institute of Botany Chinese Academy of Sciences, Kunming 650201, China.
b. University of Chinese Academy of Sciences, Beijing 100039, China.

Corresponding Authors

*xiao-dong luo

E-mail:xdluo@mail.kib.ac.cn

Tel: 86-871-65223177, Fax: 86-871-65220277

Contents

Figures	Content
S1	Bioassay and General experimental procedures
S2	^{1}H NMR spectrum of compound **1** (CD_3OD).
S3	^{13}C and DEPT spectrum of compound **1** (CD_3OD).
S4	HMBC spectrum of compound **1** (CD_3OD).
S5	HSQC spectrum of compound **1** (CD_3OD)
S6	^{1}H-^{1}H COSY spectrum of compound **1** (CD_3OD).
S7	ROESY spectrum of compound **1** (CD_3OD).
S8	HREIMS spectrum of compound **1**
S9	IR spectrum of compound **1**
S10	^{1}H NMR spectrum of compound **2** (CD_3OD).
S11	^{13}C and DEPT spectra of compound **2** (CD_3OD).
S12	HSQC spectrum of compound **2** (CD_3OD).
S13	HMBC spectrum of compound **2** (CD_3OD)
S14	^{1}H-^{1}H COSY spectrum of compound **2** (CD_3OD).
S15	ROESY spectrum of compound **2** (CD_3OD).

S16	HREIMS spectrum of compound **2**
S17	IR spectrum of compound **2**
S18	^{1}H NMR spectrum of compound **3** (CD_3OD).
S19	^{13}C and DEPT spectra of compound **3** (CD_3OD).
S20	HMBC spectrum of compound **3** (CD_3OD).
S21	HSQC spectrum of compound **3** (CD_3OD)
S22	^{1}H-^{1}H COSY spectrum of compound **3** (CD_3OD).
S23	ROESY spectrum of compound **3** (CD_3OD).
S24	HREIMS spectrum of compound **3**
S25	IR spectrum of compound **3**

S1 Bioassay and General experimental procedures

S1.1 Cytotoxicity Assay

The cytotoxic assay was performed using the MTT method, as previous method with slight modification. Briefly, human tumor cells were seeded into 96-well plates and permitted to adhere for 12 h before drug addition. For suspended cells, they were seeded immediately before drug addition with an initial density of 5 $\times 10^4$ cells/ml. Each cell line was incubated with different concentrations of the compounds for 48 h. DDP and Taxol were used as positive controls. Cell viability was measured and IC 50 values were calculated.

Table S1 Cytotoxicity of compounds **1-3** against HL-60, A-549, SMMC-7721, MCF-7 and SW480 cell lines (IC_{50} μM).

**sample**	**HL-60**	**SMMC-7721**	**A-549**	**MCF-7**	**SW480**
**1**	>40	>40	>40	>40	>40
**2**	>40	>40	>40	>40	>40
**3**	>40	>40	>40	>40	>40
**DDP(MW300)**	1.28	4.35	5.68	14.97	17.54
**Taxol**	<0.008	<0.008	<0.008	<0.008	<0.008

S1.2 General experimental procedures

General experimental procedures. Optical rotations were measured on a JASCO P-1020 digital polarimeter. IR spectra were recorded on a Bruker Tensor-27 infrared spectrophotometer with KBr pellets. ESI-MS spectra were recorded on a Bruker HTC/Esquire spectrometer. HR-ESI-MS spectra were recorded on an API Qstar Pulsar instrument. 1D and 2D NMR spectra were performed on a Bruker AVANCE III-600 spectrometer with TMS as the internal standard. Chemical shifts (δ) were expressed in ppm with reference to the solvent signals. Semi-HPLC was performed on a Waters 2489 HPLC with an Agilent ZORBAX SB-C18 (9.4 $\times$ 250 mm) column. Column chromatography (CC) was performed on silica gel (200–300 mesh, Qingdao Marine Chemical Ltd., Qingdao, China). RP-18 gel (20–45 mm, Fuji Silysia Chemical Ltd, Japan), SephadexLH-20 (Pharmacia Fine Chemical Co, Ltd, Sweden). Fractions were monitored by TLC (GF254, Qingdao Marine Chemical Ltd., Qingdao, China), and spots were visualized by Dragendorff's reagent and 10% H_2SO_4 in ethanol. GC analysis was performed on a Shimadzu GC-2010 gas chromatograph equipped with an H_2 flame ionization detector. L-rhamnose and D-glucose were purchased from J K Scientific Ltd. (Guangzhou, China).

Figure S2. ^{1}H NMR spectrum of compound **1** (CD_3OD).

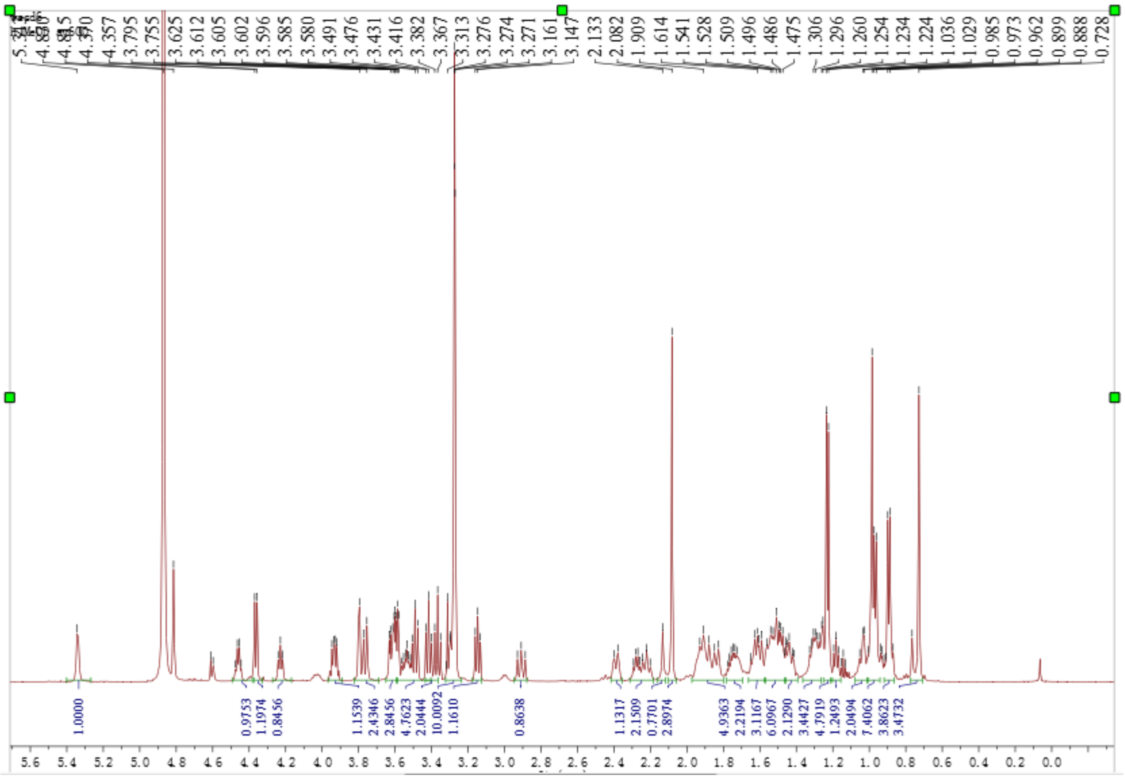

Figure S3. ^{13}C and DEPT spectrum of compound **1** (CD_3OD)

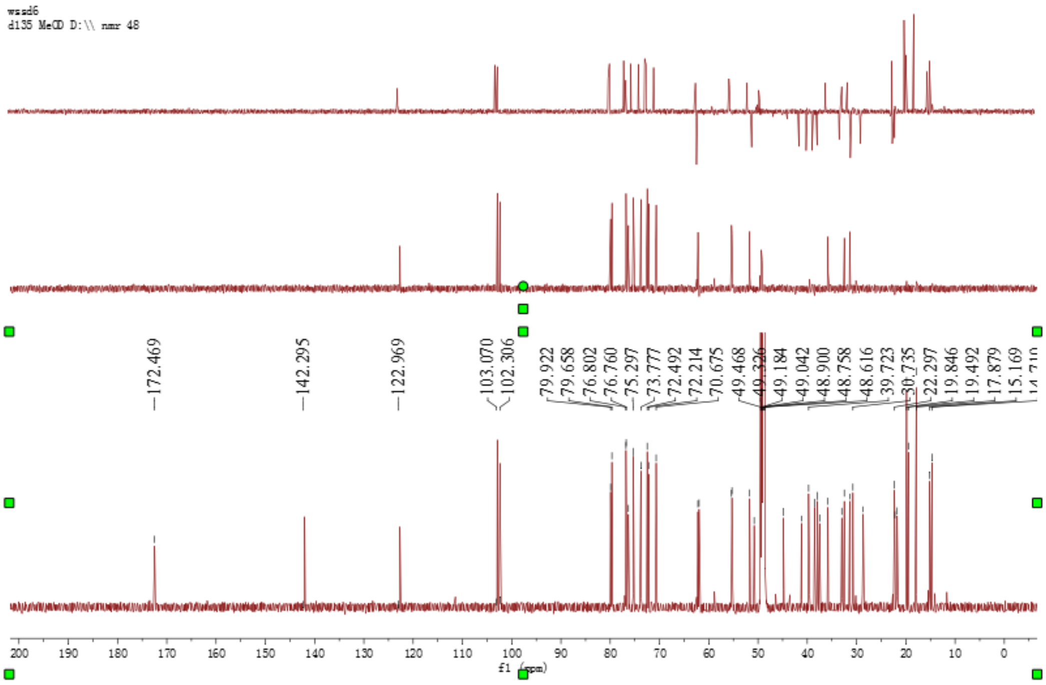

Figure S4. HMBC Spectrum of compound **1** (CD_3OD)

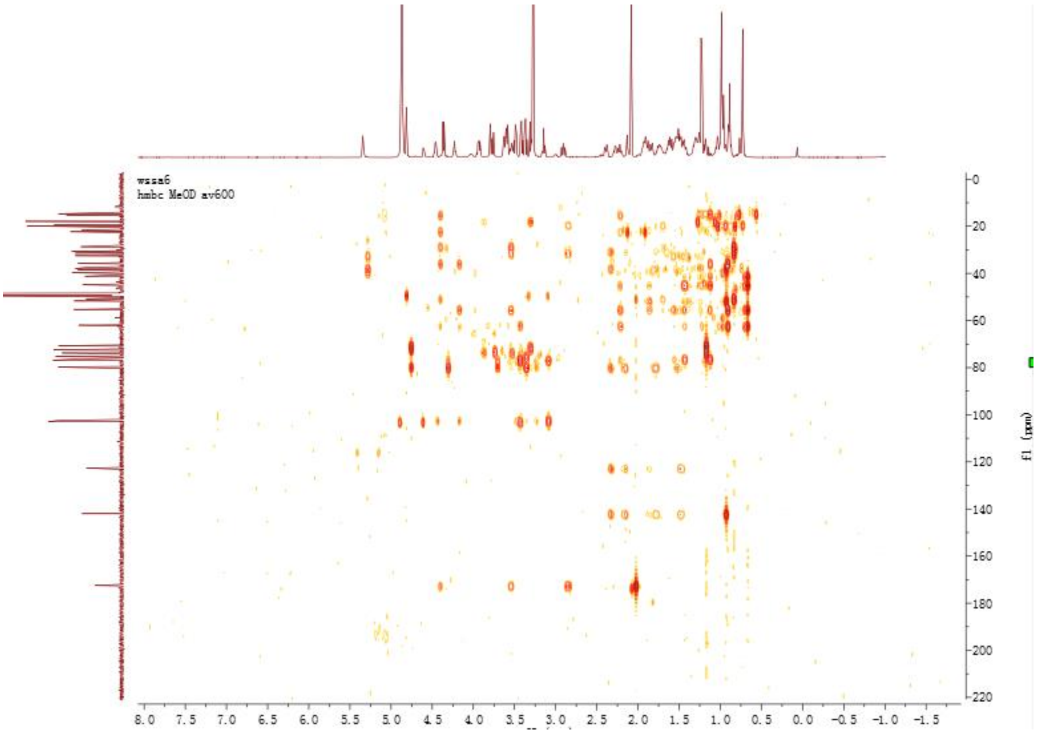

Figure S5. HSQC Spectrum of compound **1** (CD_3OD)

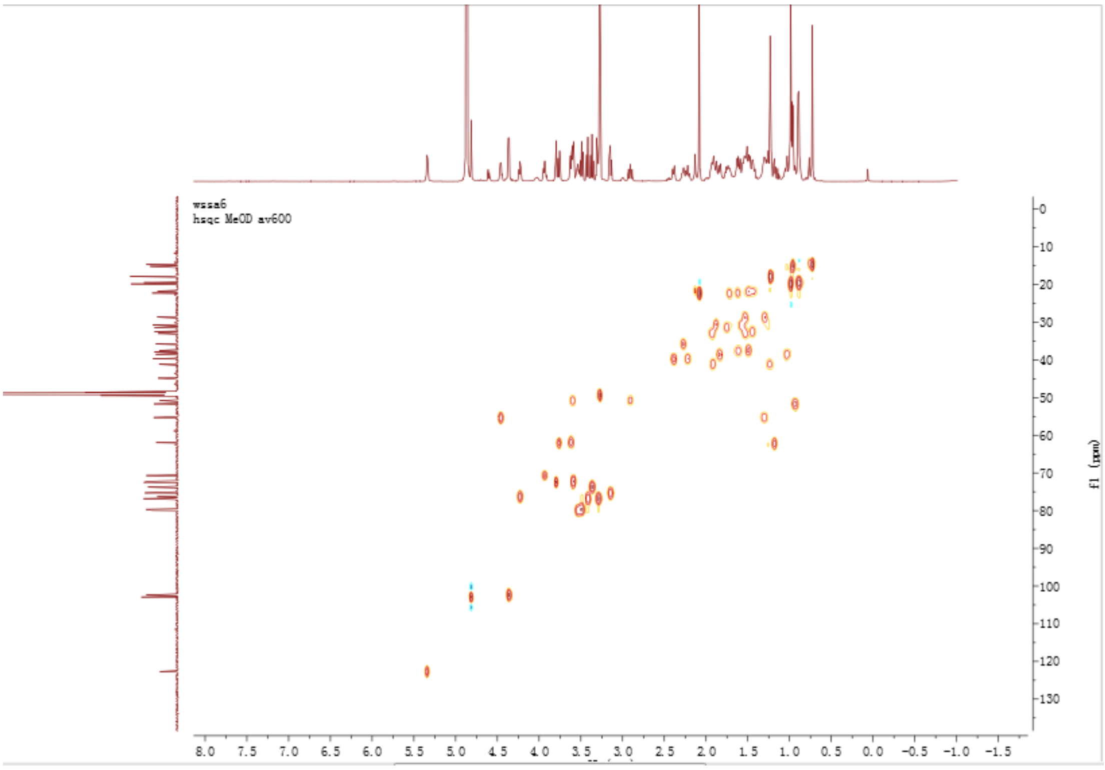

Figure S6. ^{1}H-^{1}H COSY Spectrum of compound **1** (CD_3OD)

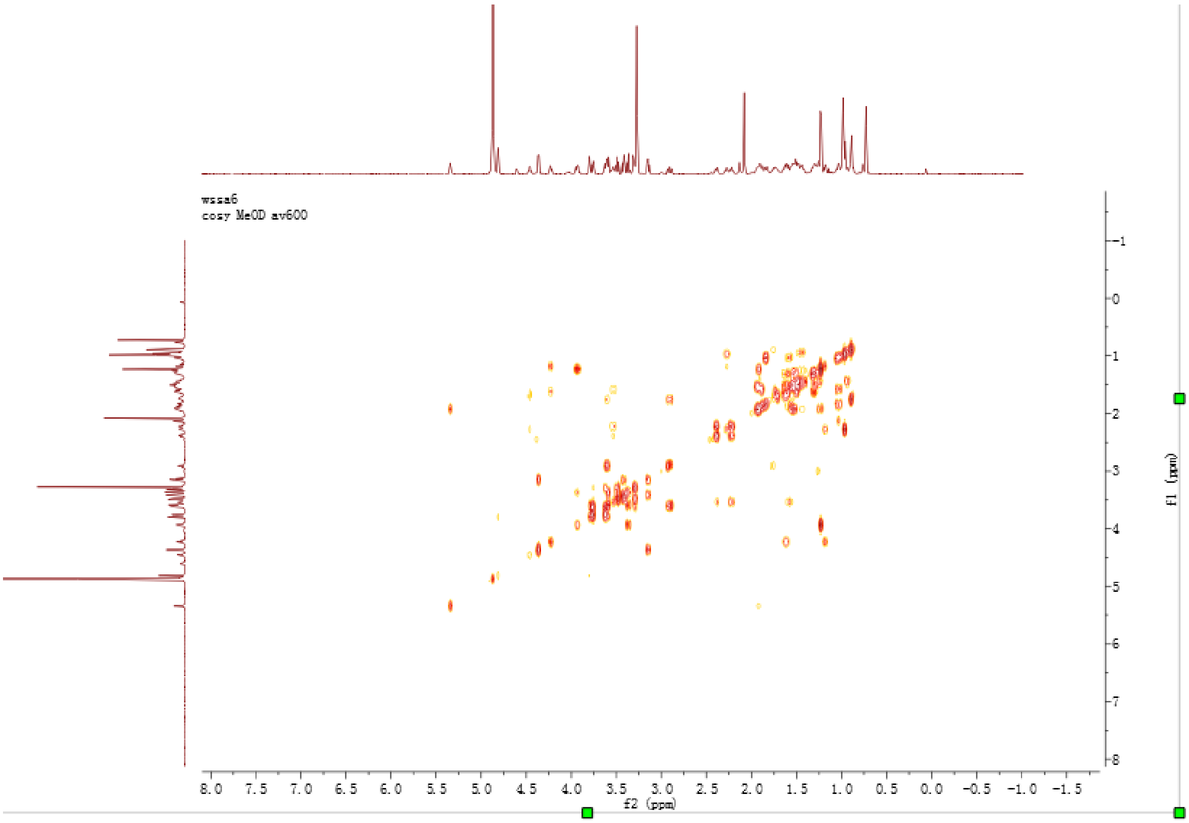

Figure S7. ROSEY Spectrum of compound **1** (CD_3OD)

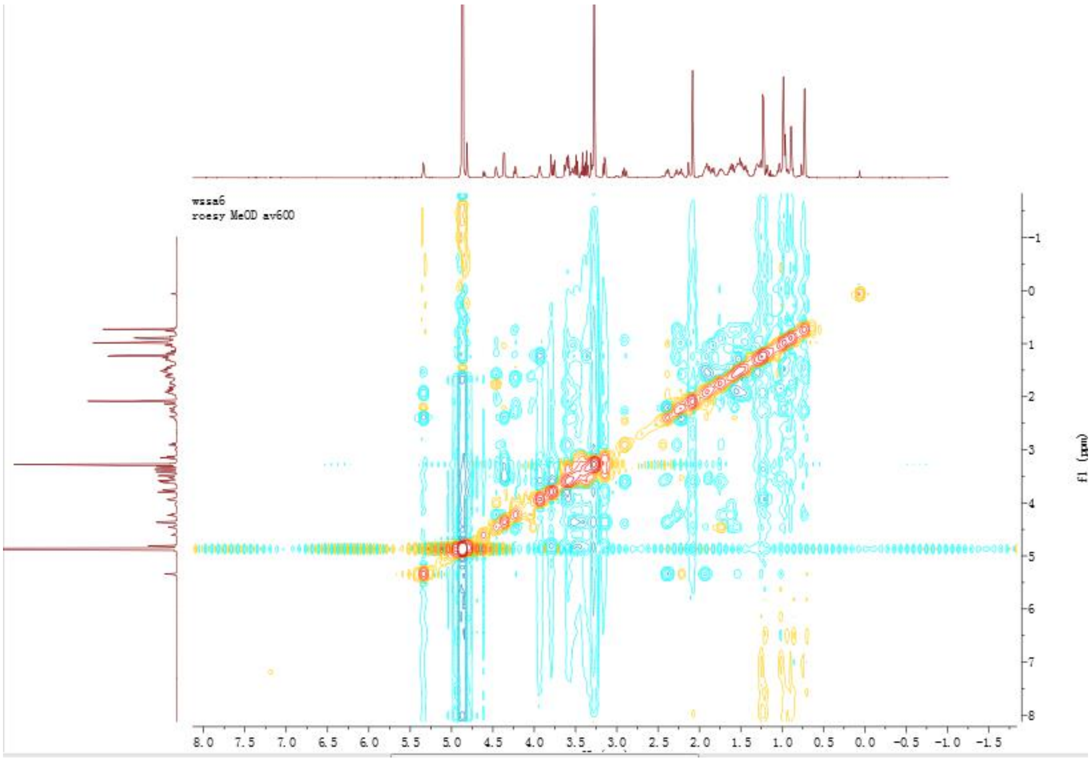

Figure S8. HRESIMS Spectrum of compound **1**

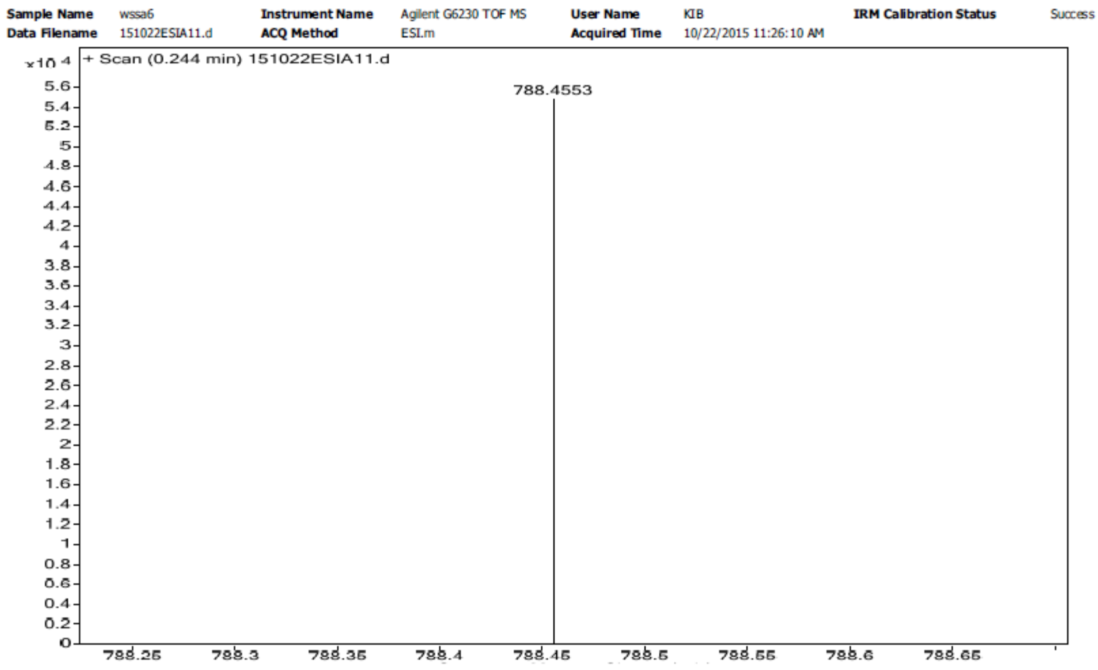

Figure S9. IR spectrum of compound **1**

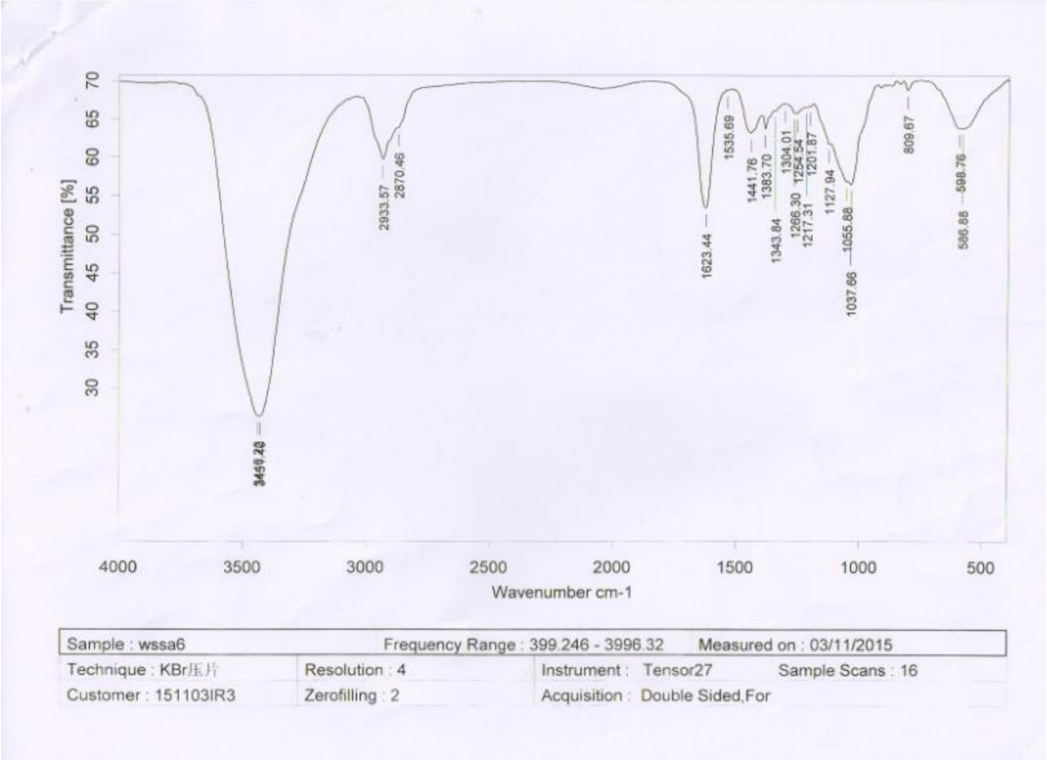

Figure S10. ^{1}H NMR spectrum of compound **2** (CD_3OD)

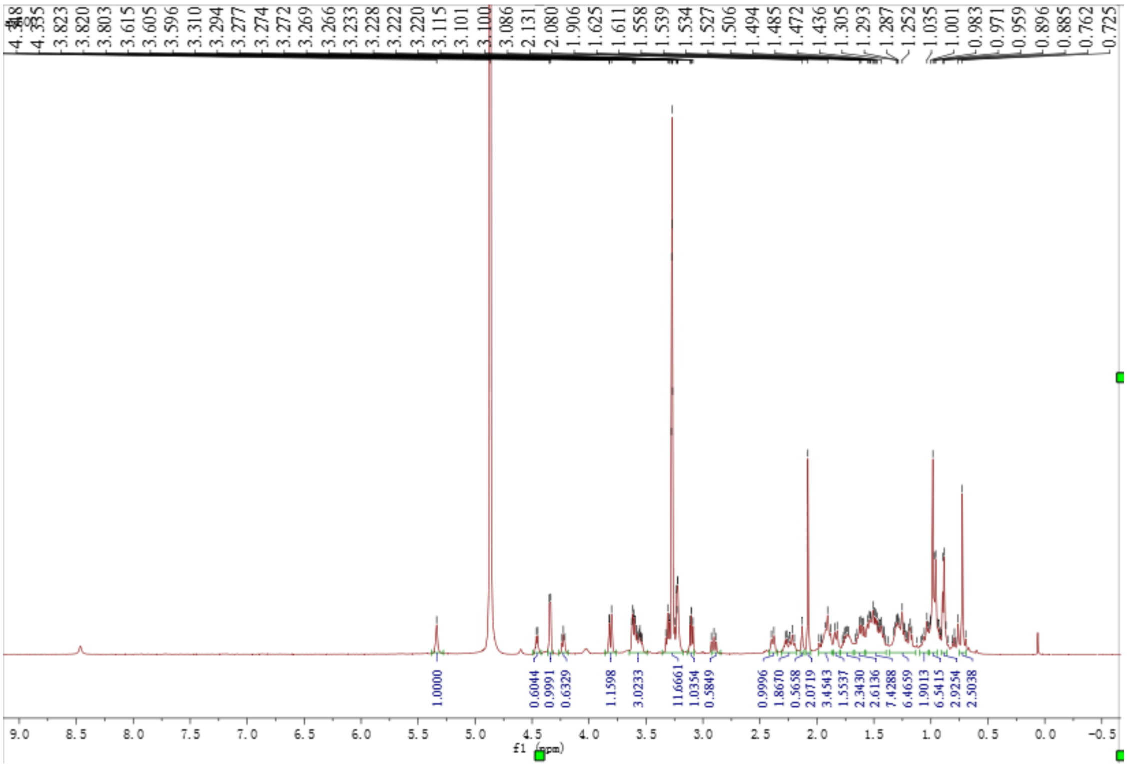

Figure S11. ^{13}C and DEPT spectrum of compound **2** (CD_3OD)

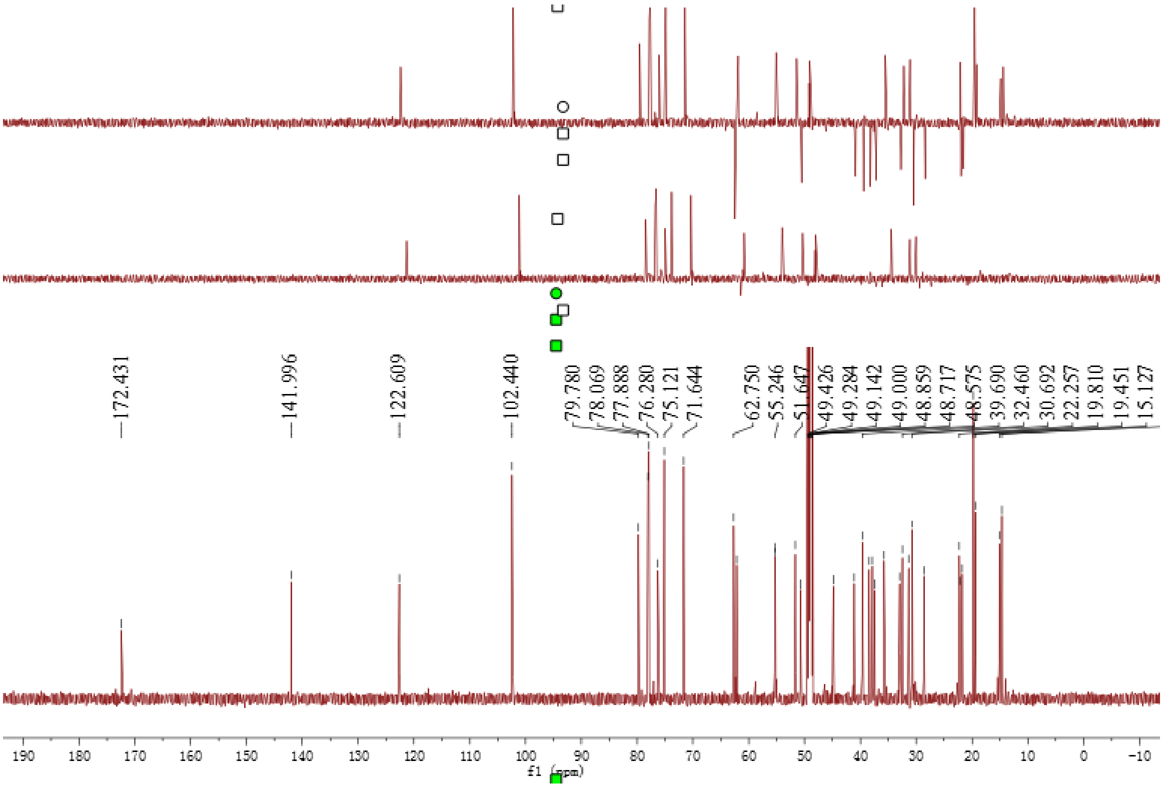

Figure S12. HSQC spectrum of compound **2** (CD_3OD)

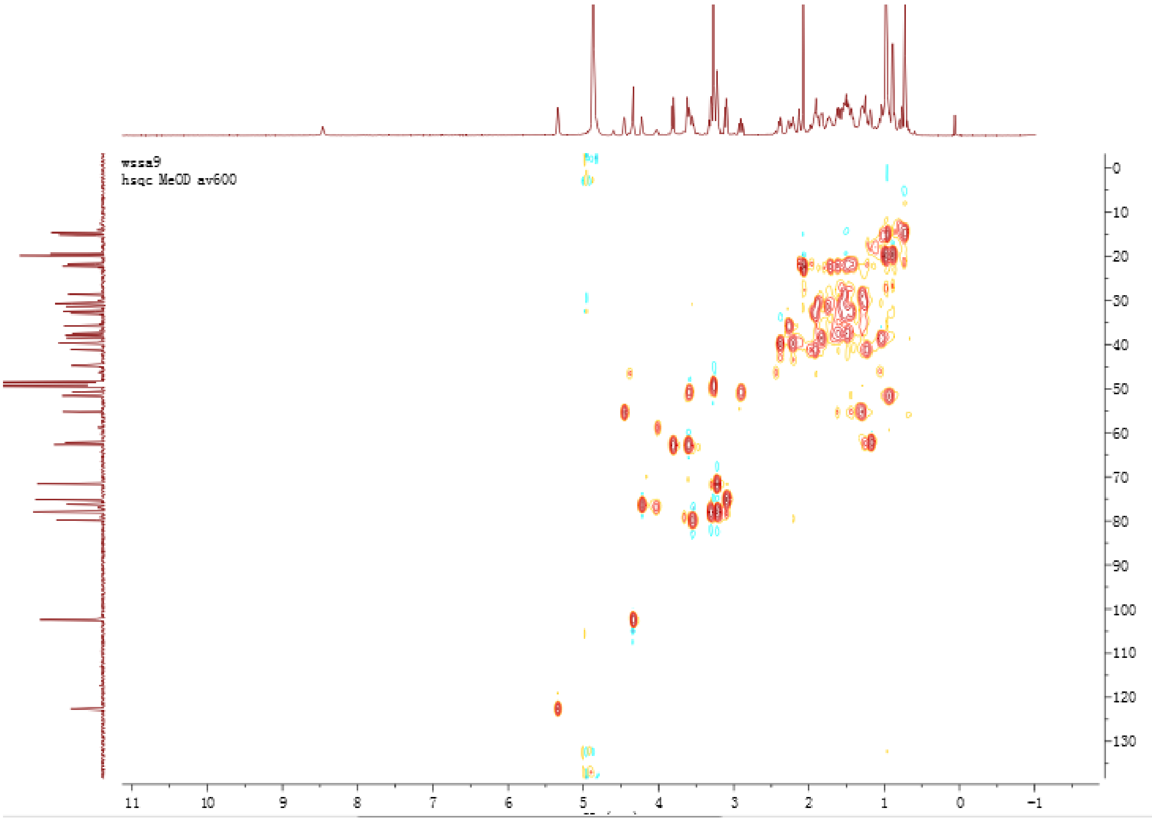

Figure S13. HMBC Spectrum of compound **2** (CD_3OD)

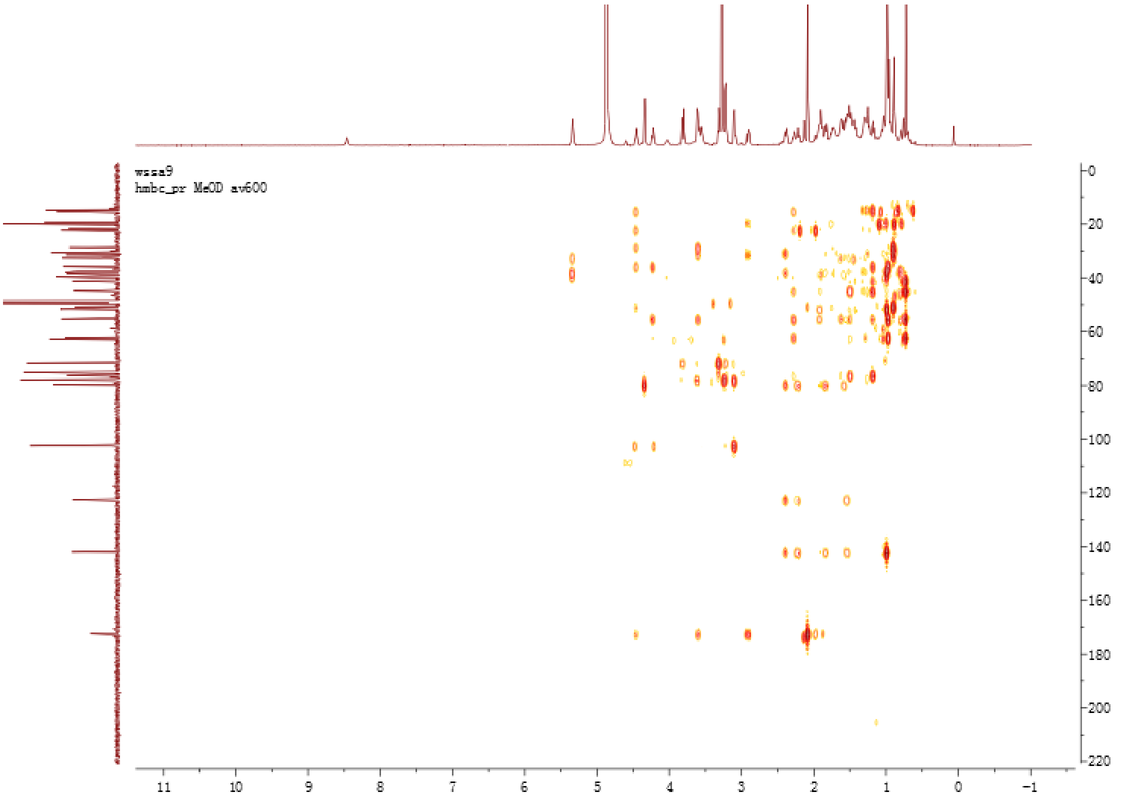

Figure S14. ^{1}H-^{1}H COSY Spectrum of compound **2** (CD_3OD)

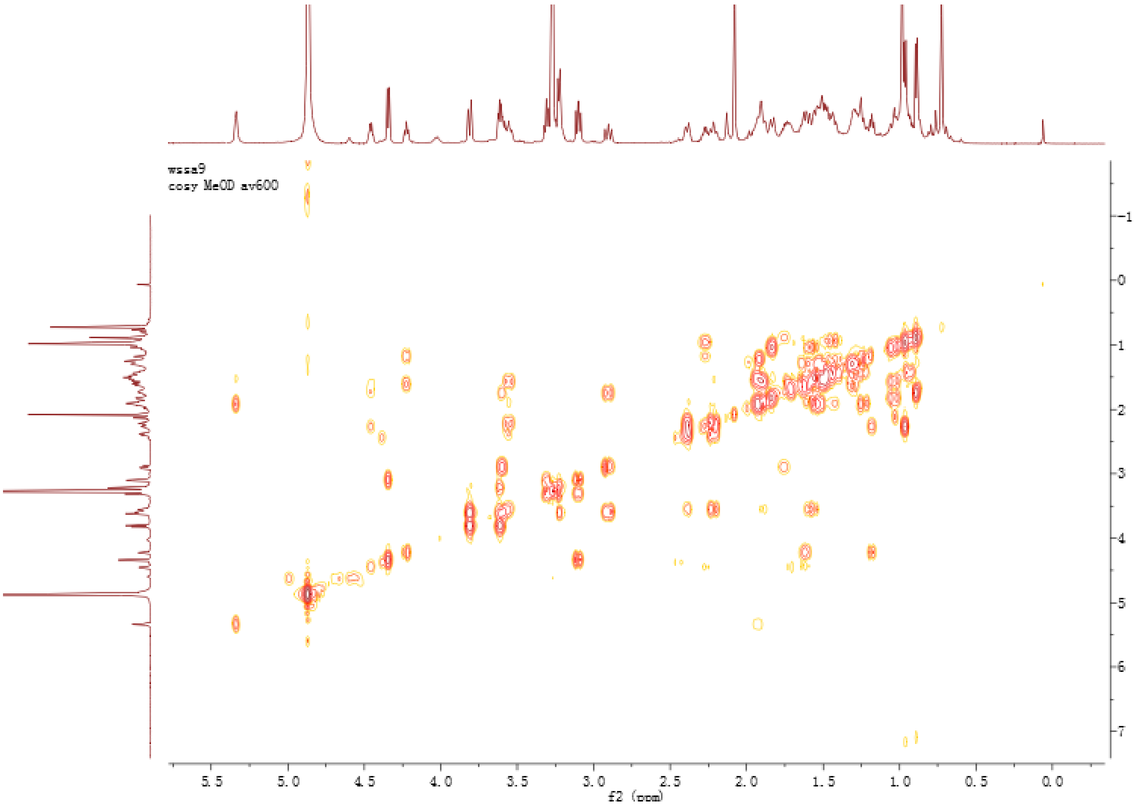

Figure S15. ROSEY Spectrum of compound **2** (CD_3OD)

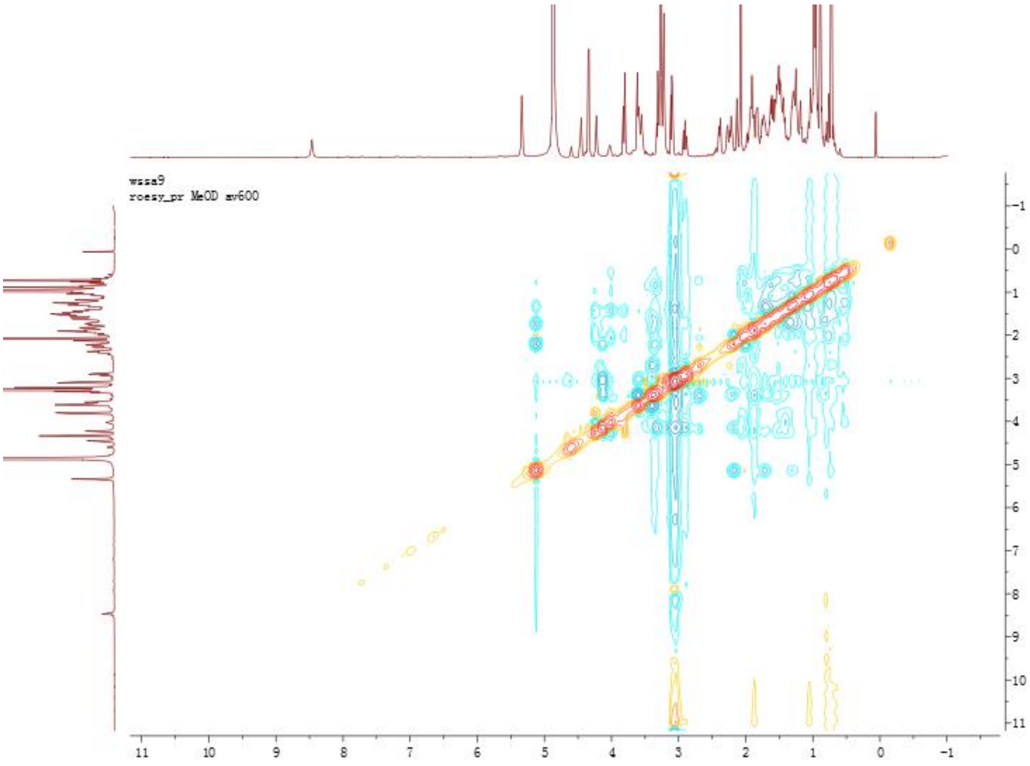

Figure S16. HRESIMS Spectrum of compound **2**

Qualitative Analysis Report

**Data Filename**	151022ESIA12.d	**Sample Name**	wssa9
**Sample Type**	Sample	**Position**	
**Instrument Name**	Agilent G6230 TOF MS	**User Name**	KIB
**Acq Method**	ESI.m	**Acquired Time**	10/22/2015 11:27:27 AM
**IRM Calibration Status**	Success	**DA Method**	ESI.m
**Comment**			

Sample Group **Info.**
Acquisition SW 6200 series TOF/6500 series
Version Q-TOF B.05.01 (B5125.2)

User Spectra

Fragmentor Voltage	Collision Energy	Ionization Mode
200	0	ESI

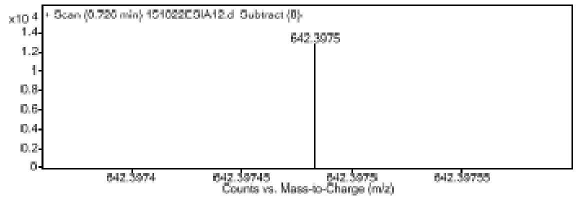

Peak List

m/z	z	Abund	Formula	Ion
116.1435	1	6515.21		
505.3333	1	2181.61		
620.413	1	11626.37		
621.4167	1	3263.45		
642.3975	1	12665.85	C35 H57 N Na O8	M+
643.4002	1	4081.38	C35 H57 N Na O8	M+
683.4272	1	1410.41		
753.308	1	1699.55		
778.3714	1	1636.28		
816.3193	1	1973.01		

Formula Calculator Element Limits

Element	Min	Max
C	0	200
H	0	400
O	5	11
Na	1	1
N	1	1

Formula Calculator Results

Formula	CalculatedMass	CalculatedMz	Mz	Diff. (mDa)	Diff. (ppm)	DBE
C35 H57 N Na O8	642.3982	642.3976	642.3975	0.3	0.5	7.5000

Figure S17. IR Spectrum of compound **2**

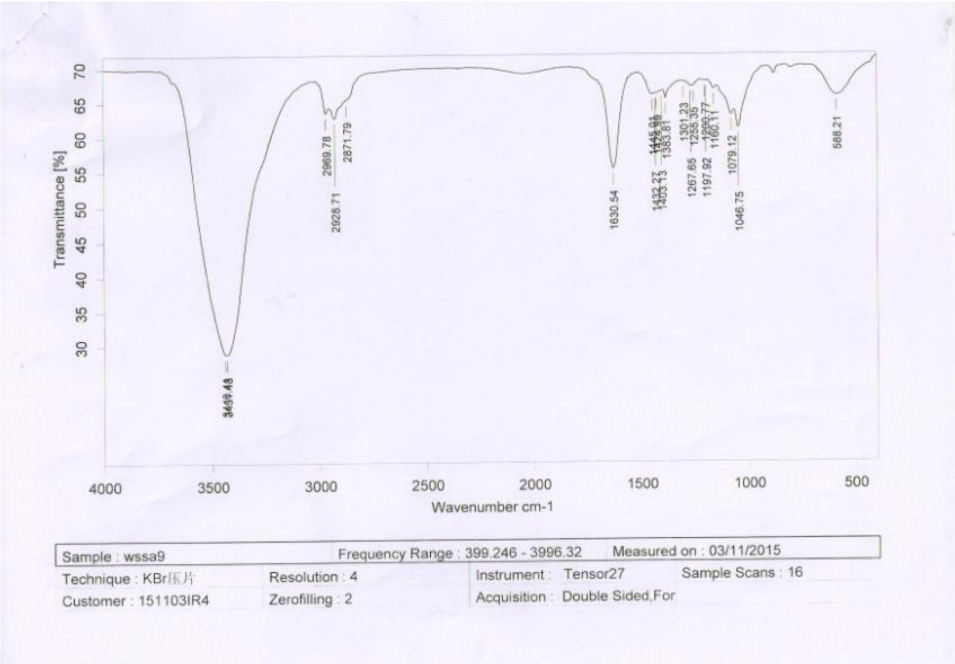

Figure S18. ^{1}H NMR spectrum of compound **3** (CD_3OD)

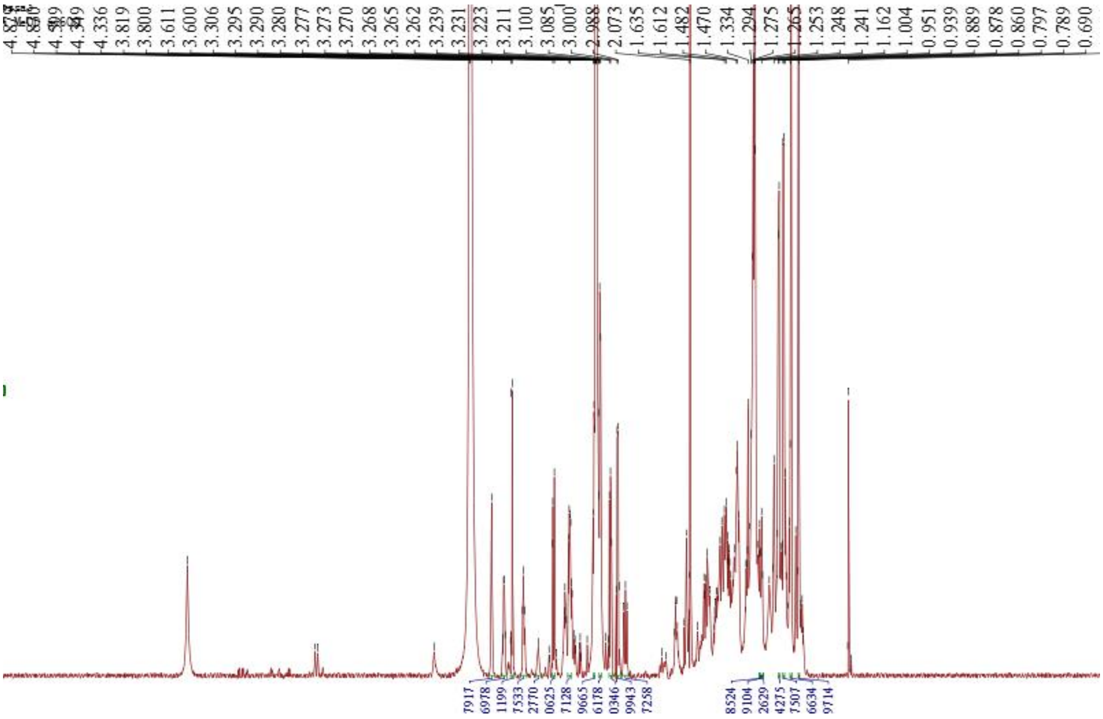

Figure S19. ^{13}C and DEPT spectrum of compound **3** (CD_3OD)

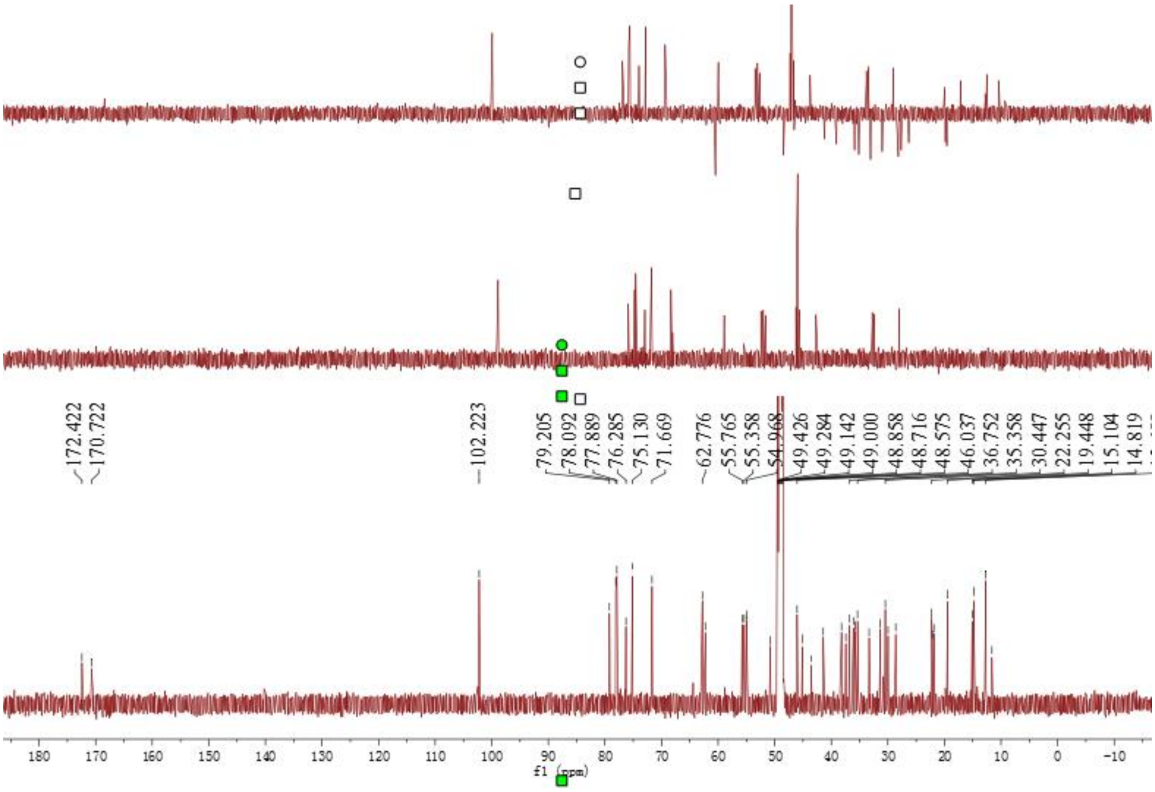

Figure S20. HMBC Spectrum of compound **3** (CD_3OD)

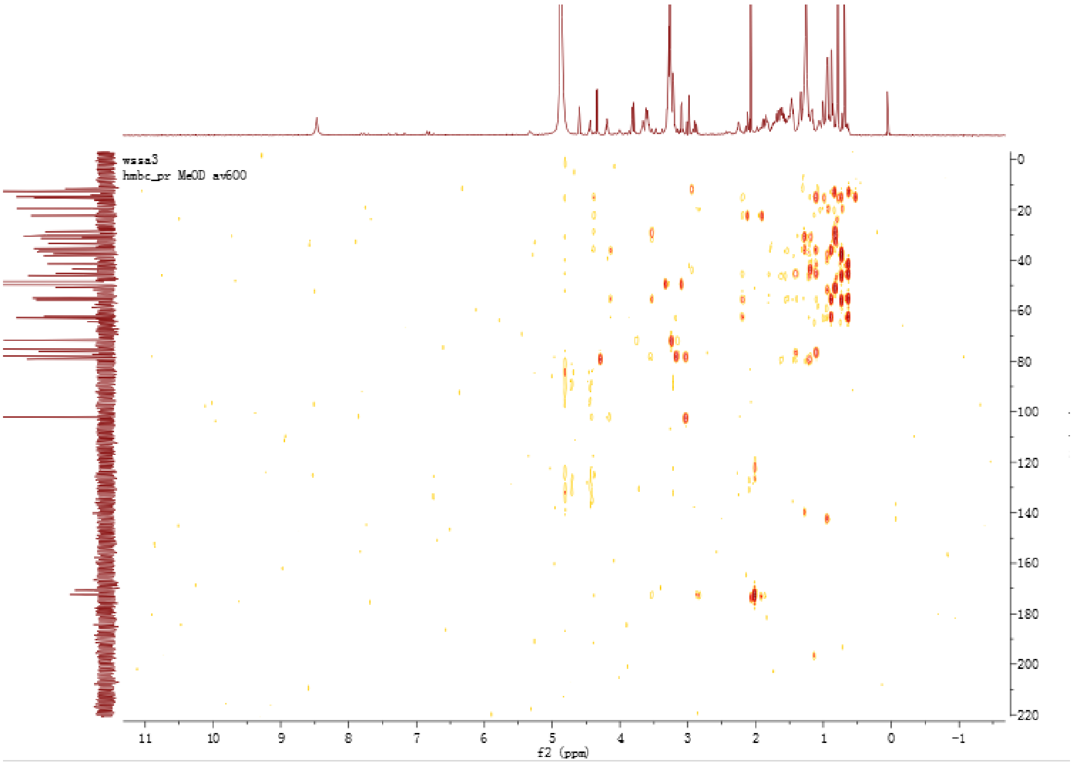

Figure S21. HSQC Spectrum of compound **3** (CD_3OD)

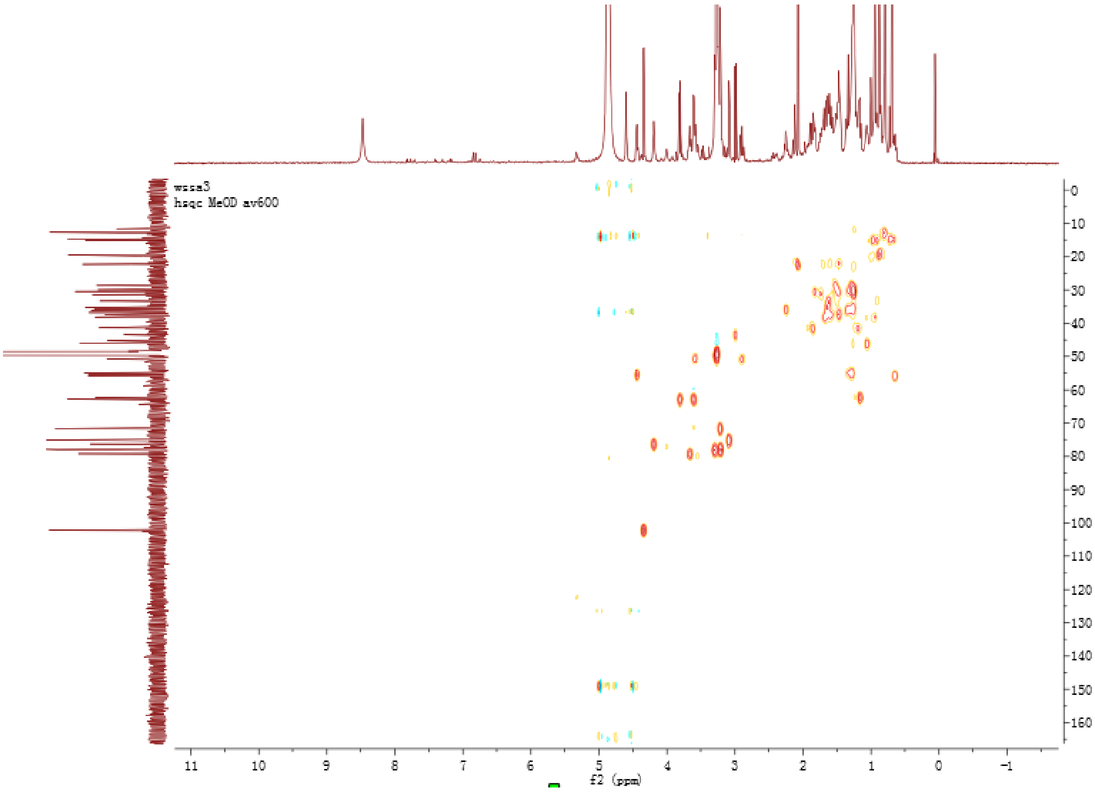

Figure S22. ^{1}H-^{1}H COSY Spectrum of compound **3** (CD_3OD)

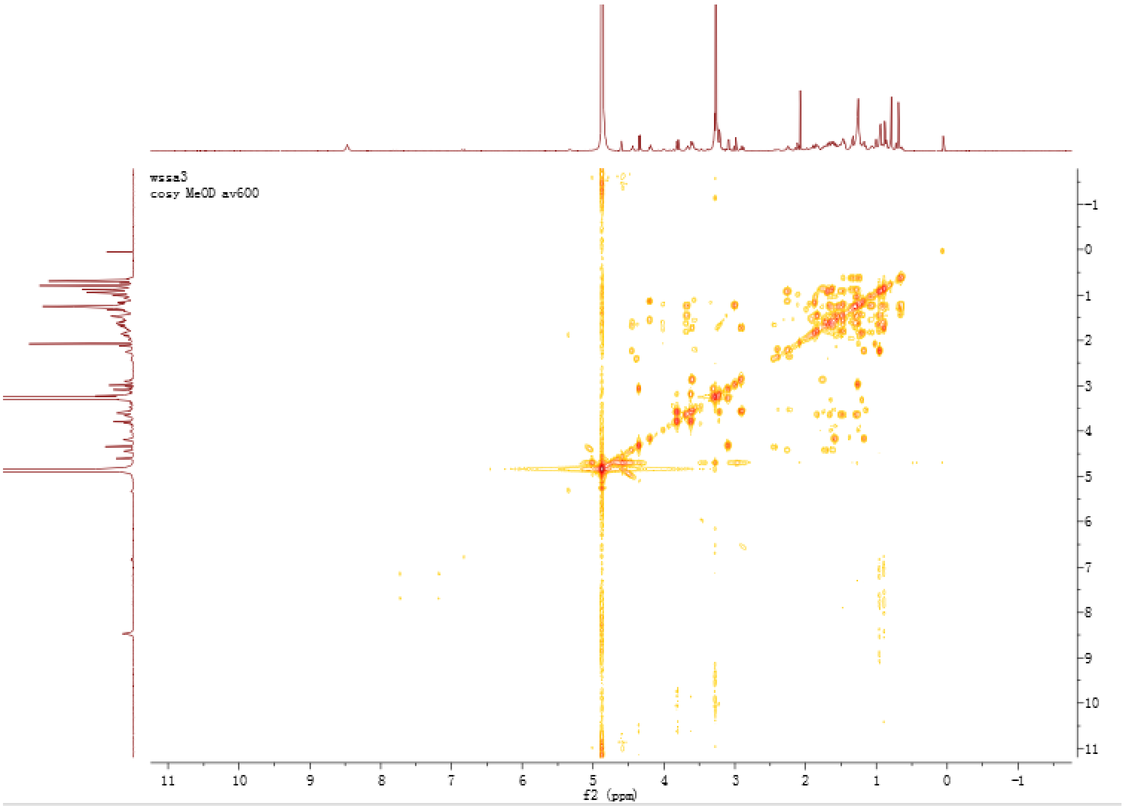

Figure S23. ROSEY Spectrum of compound **3** (CD_3OD)

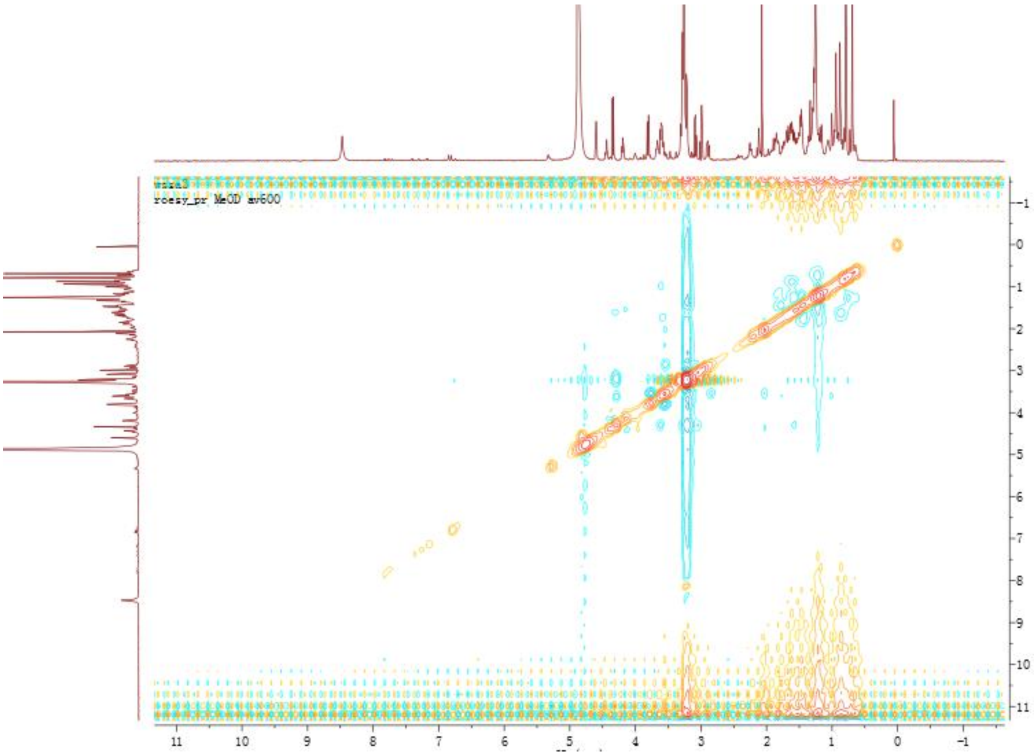

Figure S24. HRESIMS Spectrum of compound **3**

Elemental Composition Report

Page 1

Single Mass Analysis
Tolerance = 10.0 PPM / DBE: min = -10.0, max = 120.0
Selected filters: None

Monoisotopic Mass, Odd and Even Electron Ions
24 formula(e) evaluated with 1 results within limits (up to 51 closest results for each mass)
Elements Used:
C: 0-200 H: 0-400 N: 1-1 O: 7-9

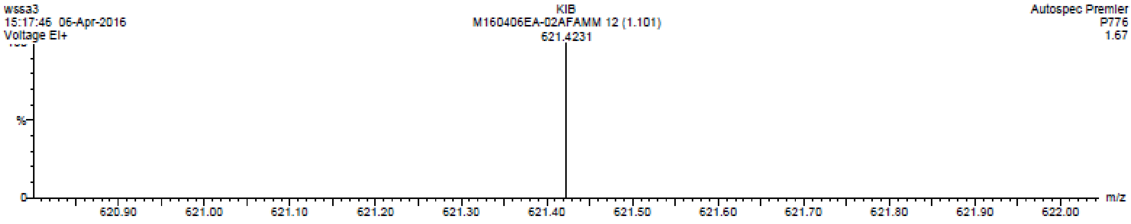

Minimum:				-10.0		
Maximum:		200.0	10.0	120.0		
Mass	Calc. Mass	mDa	PPM	DBE	i-FIT	Formula
621.4231	621.4241	-1.0	-1.6	7.0	5546025.5	C35 H59 N O8

Figure S25. IR Spectrum of compound **3** (CD_3OD)

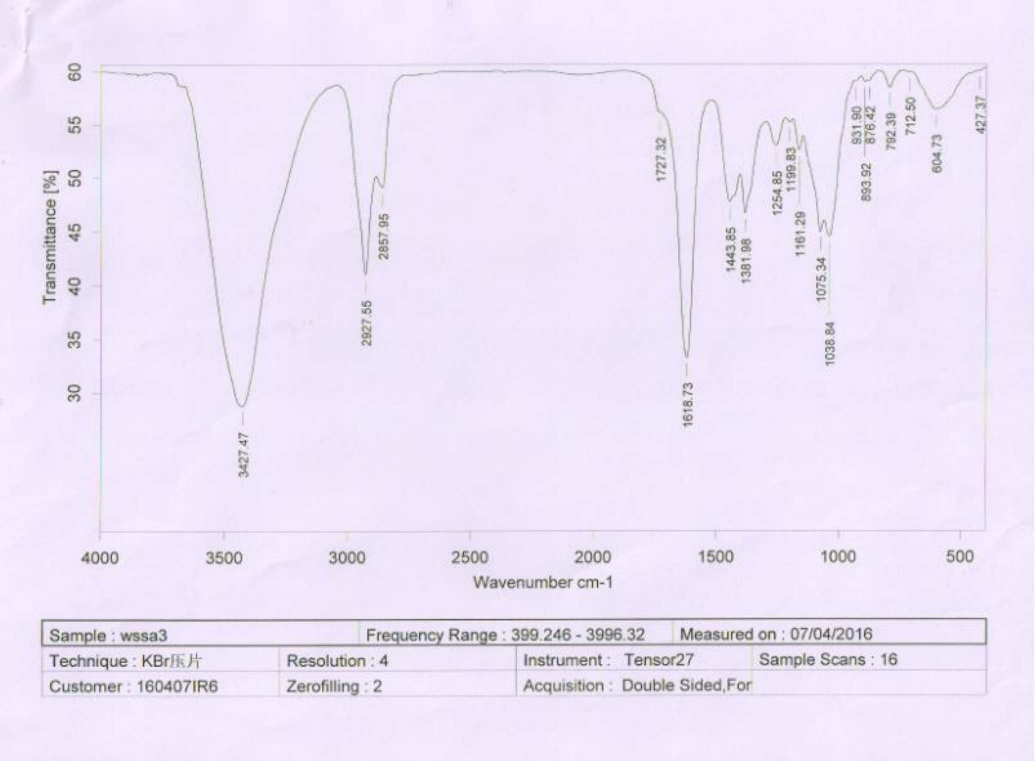

Supplement: Supplementary file 1 — Supporting Information Available: bioassay, 1D, 2D-NMR, MS and IR spectra of spiralosides A–C (1–3), these supplementary materials are available in the online version of this article and is accessible for authorized users (PDF 1341 kb) [file 13659_2016_103_MOESM1_ESM.pdf]
